# Correlation of Cleanliness among Different Bowel Segments during Colonoscopy: A Retrospective Study

**DOI:** 10.1155/2020/5363827

**Published:** 2020-03-03

**Authors:** Zhou Haibin, Zhang Xiaofeng, Yang Jianfeng

**Affiliations:** Department of Gastroenterology, Affiliated Hangzhou First People's Hospital, Zhejiang University School of Medicine, Hangzhou, China

## Abstract

**Objective:**

To analyze the correlation of intestinal cleanliness in each segment of the Boston Intestinal Preparation Scale.

**Methods:**

From February 2017 to October 2019, the data of patients who underwent colonoscopy in the Department of Gastroenterology, Hangzhou First People's Hospital, Zhejiang University School of Medicine, were collected. Statistical analysis was performed according to the Boston Intestinal Preparation Scale score, and the correlation of intestinal cleanliness in each region was obtained.

**Results:**

A total of 1739 patients were included. The overall score of BBPS was 6.77 ± 1.88. The scores of each region were 2.04 ± 0.84 (right lateral colon), 2.25 ± 0.68 (transverse colon), and 2.48 ± 0.64 (left colon). The difference between the regions was statistically significant (*P* < 0.05). The bowel cleanliness showed a gradual deterioration trend, and there was a positive correlation between colon cleanliness in each region. The accuracy of the transverse colon in predicting the right colon (AUC = 0.809) is higher than that of the left colon (AUC = 0.735), and the accuracy of predicting the cleanliness of the right colon intestinal tract by the cleanliness of the left colon intestinal tract is relatively low.

**Conclusion:**

Intestinal cleanliness gradually deteriorates from the direction of the insert. It is not reliable to predict the right side of poor cleanliness by using the left colon intestinal cleanliness (BBPS 0-1 score). It should continue to further endoscopy. When the cleanliness of the transverse colon is poor, then stopping further endoscopy is considered.

## 1. Introduction

Numerous patients who suffered from colon diseases were benefited from the invention of colonoscopy; the cleanliness of intestinal preparation has always been the key issue while the colonoscopy technology is developing. The scale of evaluation of intestinal cleanliness has also emerged continuously, with its advantages, disadvantages, and scope of application [1–4]. The Boston Bowel Preparation Scale (BBPS) [5] proposed by the Boston University has been proven to have high reliability and validity [6–8], and is widely used by digestive endoscopy workers in Europe, Korea, China, etc. It is also an indicator of intestinal cleanliness observed after a retrospective view and is related to the quality of the intestinal examination.

However, in real-world clinical scenarios, initial bowel examinations often observed that patients with unsatisfactory bowel preparation, which is difficult to decide whether to continue the colonoscopy observations or not. It might turn out that bowel preparations were worse, making colonoscopy examinations meaningless, but this would not be 100% true until the statistical confirmation. As of today, there is no literature to analyze the clinical correlation of cleanliness in various regions of the intestine, so it is still confusing for endoscope physicians.

Based on these premises, this article aims to investigate the relationship and provide a clinical statistical basis for the above confusion. To fulfill this goal, we will use BBPS, which has proven to be highly reliable and widely used in China, to analyze the correlation of intestinal cleanliness with a large number of samples in a random manner. By using these statistics, we will get reliable results as follows.

## 2. Materials and Methods

### 2.1. Research Object

The study was conducted from February 2017 to October 2019. The subjects of the study were patients who underwent colonoscopy in the Gastroenterology Hangzhou First People's Hospital affiliated to the Zhejiang University School of Medicine. Patients without abdominal surgery used polyethylene glycol electrolyte powder for intestinal preparation and had complete records and attached complete intestinal pictures were included in the study.

### 2.2. Research Methods

#### 2.2.1. Ethics

All methods and data analyses were approved by the local ethics board of Hangzhou First People's Hospital, Zhejiang University School of Medicine.

#### 2.2.2. The Boston Bowel Preparation Scale

**Figure 1:**
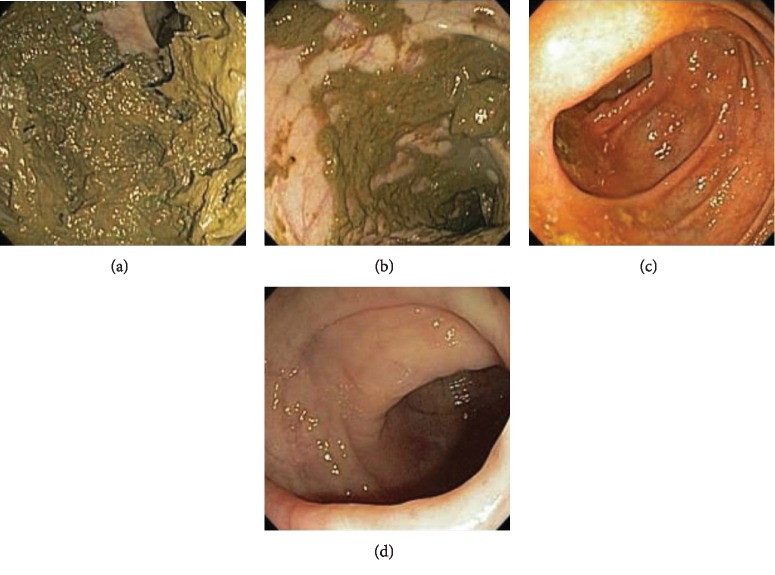
The Boston Bowel Preparation Scale (BBPS) (picture cited in Reference [5]). (a) 0 = unprepared colon segment with mucosa not seen due to solid stool that cannot be cleared. (b) 1 = portion of mucosa of the colon segment seen, but other areas of the colon segment are not well seen due to staining, residual stool, and/or opaque liquid. (c) 2 = minor amount of residual staining, small fragments of stool, and/or opaque liquid, but mucosa of colon segment is seen well. (d) 3 = entire mucosa of colon segment is seen well with no residual staining, small fragments of stool, or opaque liquid. The wording of the scale was finalized after incorporating feedback from three colleagues experienced in colonoscopy.

The Boston Bowel Preparation Scale (BBPS; suggested pronunciation “bee-bops”) was developed to limit interobserver variability in the rating of bowel preparation quality, while preserving the ability to distinguish various degrees of bowel cleanliness: right (right) lateral colon (including cecum and ascending colon), transverse colon (including liver and spleen flex), and left (left) colon (including descending colon, sigmoid colon, and rectum). According to different bowel preparation cleanliness, different evaluation scores are given in Figure 1. Each region of the colon receives a “segment score” from 0 to 3, and these scores total the total BBPS score from 0 to 9. Thus, the maximum clean BBPS score for a colon without any residual liquid is 9 and the minimum BBPS score for no colon preparation is 0. If the endoscopes discontinue surgery due to inadequate preparation, then any non-visualized proximal segments 0 points are assigned.

#### 2.2.3. Statistical Analysis

SPSS 22.0 statistical software was used for data analysis. The continuous measurement data was expressed as *x* ± *s*. The *t*-test was used for comparison between groups. The count data was expressed by the number of cases or rate (%), and the *χ*^2^ test was used for comparison between groups. *P* < 0.05 was considered statistically significant; the ROC curve test was used to predict the accuracy of the inference, and the cross-tab test was performed based on Youden's index to calculate sensitivity, specificity, misdiagnosis rate, missed diagnosis rate, positive predictive value, negative predictive value, etc. Indicators inferred prediction accuracy; Pearson's correlation analysis of colon cleanliness in each region and the use of GraphPad Prism 7.00 mapping make the results more intuitive.

#### 2.2.4. Type of Study

The clinical data of patients who underwent colonoscopy in the Gastroenterology of Hangzhou First People's Hospital affiliated to the Zhejiang University School of Medicine, from February 2017 to October 2019, were analyzed retrospectively. The cleanliness of intestinal area and the correlation between intestinal cleanliness of 1739 patients in this period were analyzed.

## 3. Result

### 3.1. Patient Characteristics

A total of 1739 cases were included in the study, including 853 males (49.1%) and 886 females (50.9%) (age 52.14 ± 13.12 years old, maximum age 87 years, minimum age 12 years; see Table 1).

**Figure 2:**
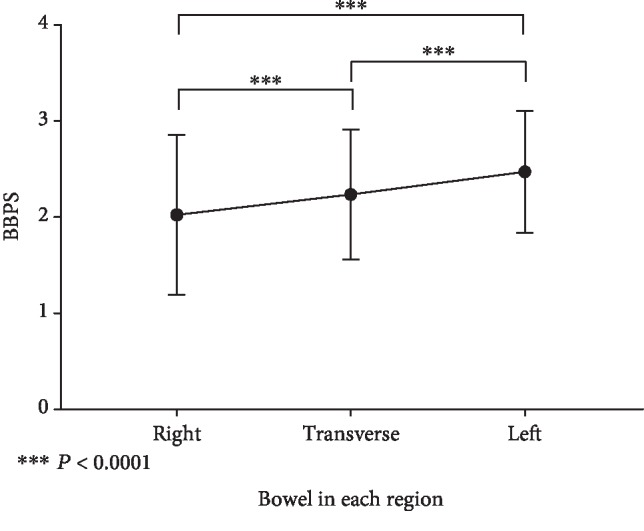
BBPS in each region bowel.

The BBPS overall score was6.77 ± 1.88, and in the right side colon (2.04 ± 0.84), and transverse colon (2.25 ± 0.68), and left colon (2.48 ± 0.64) were statistically significant (*P* < 0.05); from the ileocecal to the anus, the intestinal cleanliness shows a gradual optimization trend, as shown in Figure 2.

## 4. Correlation between Colon Cleanliness in Various Regions

Pearson's correlation analysis was performed using the right side colon, transverse colon, and left colon scores. The correlation between right and left was *r* = 0.529, *P* ≤ 0.001 < 0.05. There can be a significant positive correlation between left and right; there is a significant positive correlation between adjacent colon regions (see Table 2).

## 5. Prediction of Colon Cleanliness by Region

**Figure 3:**
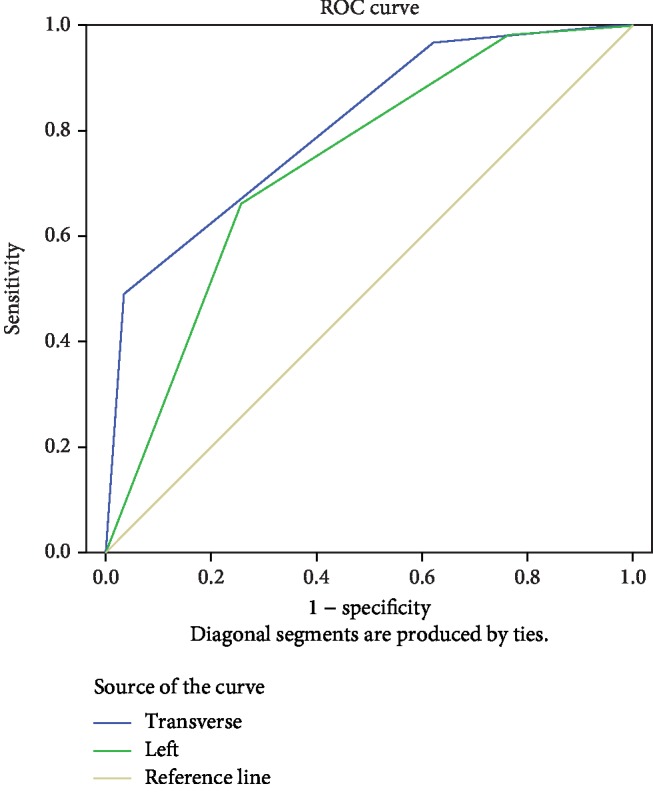
Cleanliness prediction of the right segment of the colon. Diagonal segments are produced by ties.

The right side colon score was predicted and grouped. The grouping standard is BBPS: a score of 0-1 and a value of 0 mean “poor intestinal preparation,” a score of 2-3 and a value of 1 mean “intestinal tract is ready,” the ROC test is performed with the left colon score and the transverse colon score, and the transverse colon (AUC = 0.809) is compared to the left colon score (AUC = 0.735) The case of predicting the right side colon has a better accuracy (see Figure 3 and Table 3).

**Figure 4:**
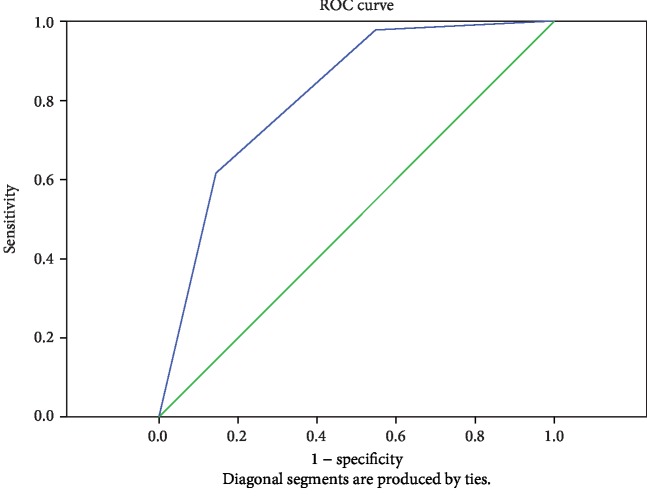
Prediction of the cleanliness of the transverse colon. Diagonal segments are produced by ties.

Left colon predicts transverse colon cleanliness, AUC = 0.814, with good accuracy (see Figure 4 and Table 4).

In this test, the score corresponding to the maximum value of Youden's index is also the value of the cut-off point of 2.5. Therefore, after grouping according to 2.5, crosschecking is performed between the regions, and then the sensitivity, specificity, misdiagnosis rate, and missed diagnosis are calculated by the formula. Indicators such as rate, positive predictive value, and negative predictive value indicate that the transverse colon predicts that the right side of the colon is relatively clean, while the left colon predicts that the right side of the colon is not highly accurate (see Table 5).

## 6. Comparison of Intestinal Cleanliness Scores by Age

The 1739 patients in this study were divided into 105 youth (0 Y-29 Y), 1091 middle aged (30 Y-59 Y), and 543 elderly (60-100 Y). The BBPS scores were 7.01 ± 1.74 points, 6.76 ± 1.93 points, and 6.74 ± 1.80 points, the total score and the cleanliness score of each area were compared, and the differences were not statistically significant (*P* > 0.05) (see Tables 6 and 7).

## 7. Discussion

Intestinal preparation plays an important role in colonoscopy and is the key to ensuring high-quality completion of colonoscopy. The 2019 European Digestive Endoscopy Society Guidelines for Intestinal Preparation [9] pointed out that poor bowel preparation could lead to poor bowel preparation. The detection rate of intestinal adenoma decreased, the failure rate of ileocecal bronchoscopy increased, the patient's pain increased, and the medical expenses were increased. The Boston Intestinal Cleanliness Scale score was also mentioned, and the total score is considered qualified if it is greater than or equal to 6.

Even if clinicians use many methods to improve intestinal cleanliness, such as WeChat, SMS, phone reminder, and laxatives, the intestinal cleanliness is improved to some extent [10–15]. Because intestinal cleanliness is the foundation of ADR, even the best new foundation must be achieved with good intestinal cleanliness [16, 17]. In the choice of intestinal cleansers, polyethylene glycol is still the most widely used at home and abroad [18], and recent meta-analysis shows sodium picosulfate/magnesium citrate with better tolerability and less frequent adverse events demonstrated non-inferior bowel cleaning efficacy than that of the polyethylene glycol [19]. In addition to active oral intestinal laxative preparations, in recent years, passive intestinal cleaning methods such as Aquanet EC-2000 have also been developed, with effects similar to oral sodium matrine sulfate and mannitol oral solutions [20, 21]. In actual clinical work, the preparation situation is not optimistic. Despite several interventions, only two-thirds of inpatients achieve adequate colon preparation before colonoscopy [22]. Even if you enter the sigmoid colonoscopy, you will find that the bowel preparation is poor (BBPS 0-1). Continued endoscopy can increase the risk of complications of colonoscopy. such as bleeding and perforation. Abandoning endoscopy will increase the risk of missed diagnosis of intestinal lesions, and there are potential legal risks. Therefore, this study provides the theoretical basis for digestive endoscopy doctors to suspend the operation, which can make the decision reasonable.

Our study found that the overall score of the BBPS score was 6.77 ± 1.88, which was slightly higher than that of the BBPS Research Center data of Boston Medical Center (6.2 ± 1.5) [5], which indicates that we have a good preparation for intestinal cleanliness in the endoscopic center. Intestinal cleanliness in the three regions is as follows: right side, 2.04 ± 0.84 points; transverse, 2.25 ± 0.68 points; and left (left) colon, 2.48 ± 0.64 points. There were significant differences among regions (*P* < 0.05). From the ileocecal part to anus, there is a trend of gradual optimization, and the difference is statistically significant. This shows that the cleanliness of the intestinal tract often deteriorates gradually after endoscopy, which may lead to failure to continue endoscopy or ineffective endoscopy and increase the risk of complications of intestinal examination. The results can guide the subsequent correlation study of colon cleanliness in each region. At the same time, the intestinal preparation requires that the excrement should be clear water or yellow without slag. This study can prove the accuracy of this viewpoint.

Our study found that there was a positive correlation between the cleanliness of the adjacent intestines in the three areas, which was consistent with the routine examination logic. At the same time, it was found that there was a positive correlation between the right colon and the left colon, which were a non-adjacent colon. This indicated that there was a theoretical support for predicting the intestinal preparation of the right colon (examination endpoint) through the left colon (examination starting point). Therefore, ROC curve was formed, and it was concluded that the accuracy of predicting the right colon (AUC = 0.809) was higher than that of the left colon (AUC = 0.735), which was consistent with the examination logic with high routine proximity accuracy. Based on Youden's index, the cross-tabulation test showed that the accuracy of left colon intestinal cleanliness prediction for right colon intestinal cleanliness was not high, while the accuracy of transverse colon prediction for right colon was high. Therefore, the conclusion of this study is that the accuracy of colon cleanliness prediction in adjacent areas is high, and there is an error in the accuracy of colon cleanliness prediction on the right side using the left colon. In clinical operation, the endoscope cannot be stopped because the intestinal cleanliness difference (BBPS 0-1 score) is found in the anus, i.e., the intestinal cleanliness difference on the right side is inferred, but if the intestinal cleanliness difference on the transverse colon (BBPS 0-1 score) is found, the endoscope can be stopped.

At the same time, due to the large sample size and large age span of the sample study, we stratified according to age group, 105 young people (0Y-29Y), 1091 middle aged (30Y-59Y), and 543 elderly (60-100Y). By comparing the differences in intestinal cleanliness among different age groups, we found that the differences were not statistically significant, suggesting that the data in this study is highly reliable and the results are not affected by age differences.

There are still some deficiencies in this study. The main reason is that endoscopy doctors in this center did not test the reliability of the Boston scoring scale. The sample size of this study is relatively small and is a single-center study. Therefore, the conclusion of this study should be tested by big data in the later period.

In summary, the intestinal cleanliness gradually deteriorates from the direction of endoscope insertion. The intestinal preparation requires that the excreta be clear water or yellow without slag. The reliability of using the left colon intestinal cleanliness difference to predict the right side poor is not good, and the endoscope should be continued. The transverse colon intestinal cleanliness difference can be considered to stop the endoscope insertion.

## Figures and Tables

**Table 1:** Characteristics of the patients.

Patients (number)	1739
Male (%)	853 (49.1%)
Age (years)	52.14 ± 13.12
Minimum age (years)	12
Maximum age (years)	87
Sedation (%)	1170 (67.3%)
Height (m)	1.68 ± 0.12
Weight (kg)	67.21 ± 12.34
BMI^∗^	23.73 ± 2.86
Comorbidities^∗∗^	
Hypertension	256 (14.7%)
Diabetes mellitus	98 (5.6%)
Coronary atherosclerotic heart disease	32 (1.8%)
Cerebral ischemic stroke	26 (1.5%)

^∗^BMI: body mass index; the values are expressed as % or mean ± SD. ^∗∗^Comorbidities: medical history provided by patient.

**Table 2:** Correlation between regions of the colon.

	Right	Transverse	Left
Right			
*r*	1	0.741^∗∗^	0.529^∗∗^
*P*		0.000	0.000
Transverse			
*r*	0.741^∗∗^	1	0.639^∗∗^
*P*	0.000		0.000
Left			
*r*	0.529^∗∗^	0.639^∗∗^	1
*P*	0.000	0.000	

**Table 3:** Area under the predicted curve of the right side colon (AUC).

Test result variable	Area	Standard error	Progressive sig.	Asymptotic 95% confidence interval	
				Lower limit	Upper limit
Transverse	0.809	0.011	0.000	0.787	0.831
Left	0.735	0.014	0.000	0.707	0.764

Test result variable: transverse, left. There is at least one knot between the positive and negative actual state groups. Statistics may vary.

**Table 4:** Area under the predicted curve of the transverse colon (AUC).

Test result variable	Area	Standard error	Progressive sig.	Asymptotic 95% confidence interval	
				Lower limit	Upper limit
Left	0.814	0.018	0.000	0.779	0.848

Test result variable: left has at least one knot between the positive and negative actual state groups. Statistics may vary.

**Table 5:** Cross-sectional test between each colon area.

Parameter	Item result (%)		
	L prediction R	T prediction R	L prediction T
Sensitivity	43.67	66.16	37.10
Specificity	100.00	74.31	99.52
False positive rate (misdiagnosis rate)	0.00	25.69	0.48
False negative rate (missing rate)	56.33	33.84	62.90
Authenticity (accuracy)	57.79	68.20	44.57
Prevalence	74.93	74.93	88.04
Positive predictive value	100.00	88.50	99.82
Negative predictive value	37.26	42.35	17.69
Positive LR	—	257.53	7716.79
Negative LR	56.33	45.54	63.20

**Table 6:** Comparison of intestinal cleanliness scores by age.

ANOVA		Square sum	df	Mean square	*F*	Significance
BBPS	Between groups	6.705	2	3.353	0.949	0.387
	Within groups	6131.973	1736	3.532		
	Total	6138.679	1738			

Right	Between groups	2.092	2	1.046	1.496	0.224
	Within groups	1213.698	1736	0.699		
	Total	1215.790	1738			

Transverse	Between groups	0.708	2	0.354	0.766	0.465
	Within groups	802.966	1736	0.463		
	Total	803.675	1738			

Left	Between groups	0.711	2	0.355	0.872	0.418
	Within groups	707.605	1736	0.408		
	Total	708.315	1738			

**Table 7:** Comparison of intestinal cleanliness scores by age.

LSD							
Dependent variable	Age (*I*)	Age(*J*)	Mean difference (I − J)	Standard error	Significance	95% confidence interval	
						Lower limit	Upper limit
BBPS	Youth	Middle aged	0.252	0.192	0.190	-0.13	0.63
		Elderly	0.271	0.200	0.176	-0.12	0.66
	Middle aged	Youth	-0.252	0.192	0.190	-0.63	0.13
		Elderly	0.020	0.099	0.843	-0.17	0.21
	Elderly	Youth	-0.271	0.200	0.176	-0.66	0.12
		Middle aged	-0.020	0.099	0.843	-0.21	0.17

Right	Youth	Middle aged	0.148	0.085	0.084	-0.02	0.32
		Elderly	0.138	0.089	0.121	-0.04	0.31
	Middle aged	Youth	-0.148	0.085	0.084	-0.32	0.02
		Elderly	-0.009	0.044	0.832	-0.10	0.08
	Elderly	Youth	-0.138	0.089	0.121	-0.31	0.04
		Middle aged	0.009	0.044	0.832	-0.08	0.10

Transverse	Youth	Middle aged	0.085	0.069	0.219	-0.05	0.22
		Elderly	0.073	0.073	0.312	-0.07	0.22
	Middle aged	Youth	-0.085	0.069	0.219	-0.22	0.05
		Elderly	-0.012	0.036	0.734	-0.08	0.06
	Elderly	Youth	-0.073	0.073	0.312	-0.22	0.07
		Middle aged	0.012	0.036	0.734	-0.06	0.08

Left	Youth	Middle aged	0.018	0.065	0.778	-0.11	0.15
		Elderly	0.059	0.068	0.383	-0.07	0.19
	Middle aged	Youth	-0.018	0.065	0.778	-0.15	0.11
		Elderly	0.041	0.034	0.222	-0.02	0.11
	Elderly	Youth	-0.059	0.068	0.383	-0.19	0.07
		Middle aged	-0.041	0.034	0.222	-0.11	0.02

## Data Availability

The data used to support the findings of this study are available from the corresponding author upon request.
